# P335: Situational analysis of patient safety national device and risk management to healthcare procedures in Ivory Coast

**DOI:** 10.1186/2047-2994-2-S1-P335

**Published:** 2013-06-20

**Authors:** OA Oyourou, M Adeoti

**Affiliations:** 1 National Institute of Public Health, Côte d'Ivoire; 2 University of Cocody, RIPAQS, Abidjan, Côte d'Ivoire

## Introduction

Patient safety and risk management in health care are major public health issues in African countries and particularly in Côte d'Ivoire who engaged in the global dynamics of systems strengthening health.

## Objectives

To analyze the profile of the patient safety and risk management situation in Côte d'Ivoire.

## Methods

This is a 2 months-prospective study conducted at headquarters, 7 national public institutions and 12 health facilities in the district of Abidjan. It focused on 69 items distributed by the WHO components of the strategy of strengthening the health system assessed according to compliance with a basic reference value of 60%.

## Results

The overall compliance rate average is 46% reflecting an inadequate level of safety in health care in Côte d'Ivoire for the 69 items evaluated on the eight components: Leadership and governance for health care benefits, human resources for health, health financing, health information systems, health technologies; Community Ownership and Participation, Partnerships for Health Development, and Health Research. Thus, the average compliance rate of Leadership and Governance (64%) characterized by development and adoption of national policy document on patient safety and risk management, community involvement (60%), health technologies (63.6%) reflect an acceptable security. An inadequate level of safety is observed on service delivery (45%), human resources (42%), health information (40%), while unacceptable safety is recognized on components for evaluation care practices (20%), finance (16%), health research (33%).

## Conclusion

The health system in Côte d'Ivoire has a low level of compliance with respect to requirements for patient safety resulting in an increased risk of serious adverse events.

## Disclosure of interest

None declared

